# Efficacy and Safety of Hypofractionated Radiotherapy for the Treatment of Newly Diagnosed Glioblastoma Multiforme: A Systematic Review and Meta-Analysis

**DOI:** 10.3389/fonc.2019.01017

**Published:** 2019-10-14

**Authors:** Guixiang Liao, Zhihong Zhao, Hongli Yang, Xianming Li

**Affiliations:** ^1^Department of Radiation Oncology, Second Clinical Medicine Centre, Shenzhen People's Hospital, Jinan University, Shenzhen, China; ^2^Department of Nephrology, Second Clinical Medicine Centre, Shenzhen People's Hospital, Jinan University, Shenzhen, China

**Keywords:** glioblastoma multiforme, hypofractionated radiotherapy, conventional fraction radiotherapy, brain tumors, gliomas, radiochemotherapy

## Abstract

**Background:** Hypofractionated radiotherapy (HFR) is sometimes used in the treatment of glioblastoma multiforme (GBM). The efficacy and safety of HFR is still under investigation. The aim of this systematic review and meta-analysis was to provide a comprehensive summary of the efficacy and safety of HFR, and to compare the efficacy and safety of HFR and conventional fraction radiotherapy (CFR) for the treatment of patients with GBM, based on the results of randomized controlled trials (RCTs).

**Methods:** A literature search was conducted to identify Phase II and III trials o comparing the efficacy and safety of HFR and CFR. Study selection, data extraction, and quality assessment, were conducted by two independent researchers. The analysis was performed using RevMan 5.3 and Stata 12.0.

**Results:** Sixteen Phase II and III trials were included in the systematic review, and four RCTs were included in the meta-analysis. Participants treated with HRF and CRF had comparable overall survival (OS) (hazard ratio [HR]: 0.94, 95% confidence interval [CI]: 0.72–1.22, *P* = 0.64) and progression-free survival (PFS) (HR: 1.09, 95% CI: 0.60–1.95, *P* = 0.79), and similar rates of adverse events. However, in participants aged >70 years, those who received HFR had a higher OS than those who received CFR (HR: 0.59, 95% CI: 0.37–0.93, *P* = 0.02).

**Conclusions:** HRF is efficacious and safe for the treatment of GBM. In individuals aged >70 years, treatment with HRF is superior to CFR in terms of OS. The role of HFR in the treatment of GBM in younger individuals and those with good prognostic factors requires further research.

## Introduction

### Rationale

Globally, glioblastoma multiforme (GBM) is one of the most common malignant neoplasms and it generally has a poor prognosis (1). Despite advances in the management of GBM, the median overall survival (OS) is <18 months (2, 3). The standard treatment of GBM is to provide 60 Gy of fraction radiotherapy, with 1.8–2.0 Gy per fraction, over a period of 6 weeks with concurrent temozolomide (TMZ) (3). The provision of radiation therapy may be associated with better survival outcomes compared to the provision of supportive care alone. However, the optimized dose and fraction of radiation therapy has not been determined.

In newly diagnosed patients with GBM, indicators of more favorable prognosis include age <70 years, maximal safe resection of the tumor, followed by conventional fraction radiotherapy (CFR) concurrent with adjuvant TMZ (4). Hypofractionated radiotherapy (HFR) delivers more than five fractions with a higher dose per fraction (>2 Gy) and fewer exposure times (4). It has not been established whether the radiotherapy dose intensification of HFR improves the prognosis or the quality of life (5–9). Inhibiting tumor repopulation and shorten the treatment duration are potential advantages of HFR (10–12). Furthermore, a shorter treatment time might be associated with a better quality of life in patients with poorer prognosis (13, 14). HFR is generally considered to be effective and safe, and is associated with limited morbidity (15, 16).

However, one study found that patients with GBM who received HFR had a poorer survival outcomes than those who received CFR, based on an analysis of American National Cancer Database (17). In addition, the effect of HFR on outcome in patients with good prognostic factors is still under investigation.

### Objectives and Research Question

Therefore, we conducted a systematic review and meta-analysis to determine the efficacy and safety of HFR f, and the relative efficacy and safety of HFR compared to conventional fraction radiotherapy (CFR), for the treatment of patients with GBM, based on the results of randomized controlled trials (RCTs).

## Methods

### Study Design

We conducted a systematic review and meta-analysis according to the Preferred reporting Items for Systematic Reviews and Meta-Analyses (PRISMA) criteria (18).

The review considered Phase II or Phase III trials published as full articles. Retrospective studies were excluded.

### Participants, Interventions, and Comparator

Patients aged >16 years with GBM confirmed by pathology. Patients with a recurrence following treatment were excluded.

#### Intervention

**Treatment group:** Patients who received HFR, with or without concurrent chemotherapy.

**Control group:** Patients who received CFR, with or without concurrent chemotherapy.

#### Outcomes

**Primary outcomes:**

Overall survival (OS).

Progression-free survival (PFS).

**Secondary outcomes:**

Adverse effects of treatment, quality of life and neurocognitive function.

### Search Strategy and Data Sources

The PubMed, Cochrane Library and EMBASE databases were searched from their inception to April 2019. The MeSH terms and keywords used the words “hypofractionated,” “glioma,” and “radiotherapy.” The search method in PubMed was used the following algorithm in all fields: (hypofractionated OR short course OR abbreviated) AND (radiation OR radiotherapy) AND (glioma^*^ OR glioblastoma^*^ OR astrocytoma^*^ OR oligodendroglioma^*^ OR oligoastrocytoma^*^). The references provided in the studies identified was checked for the potential additional studies meeting the inclusion criteria.

### Study Selection and Data Extraction

The articles identified were assessed by two reviewers (GL and ZZ), independently. Any disagreement was resolved by discussion among the researchers.

The data were extracted independently by the two reviewers. The following information on each study was checked and extracted.

1. Authors, year of publication, sample size, treatment methods, and the duration of follow-up.
2. OS and PFS.
3. Adverse events, quality of life, and neurocognitive function

Cochrane tools were used to assess the quality of each of the studies that met the inclusion criteria for meta-analysis (19). The following items were evaluated: selection bias, performance bias, detection bias, attrition bias, reporting bias, and other sources of bias.

### Data Analysis

The analysis was conducted using RevMan, Version 5.3 (Nordic Cochrane Centre), and Stata, Version 12.0 (StataCorp LLC, College Station, TX, USA). Survival outcomes were measured using hazard ratios (HRs) with their 95% confidence intervals (CIs). Adverse events were measured using odds ratios (ORs) and their 95% CIs.

Heterogeneity was evaluated using *I*^2^, and values of 25, 50, and 75% were considered low, moderate, and high, respectively (20). If *I*^2^ was <25%, data analysis was conducted using a fixed-effects model; otherwise, a random-effects model was used. *P*-values < 0.05 were regarded as statistically significant. In addition, we conducted subgroup analyses (by ages) and sensitivity analyses (by omitting any single study). Publication bias was evaluated using Egger's test and Begg's test (21).

## Results

### Study Selection and Characteristics

**Figure 1:**
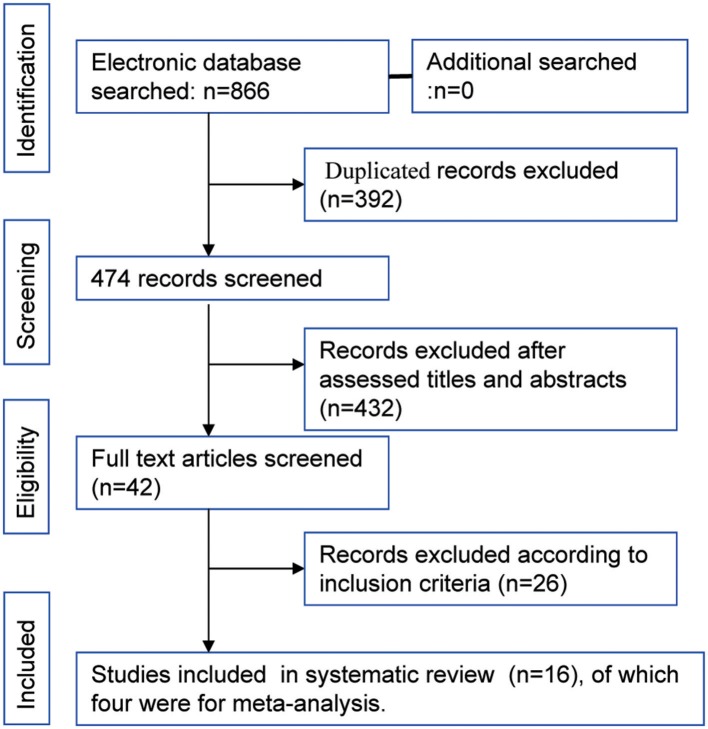
The study selection process.

**Figure 2:**
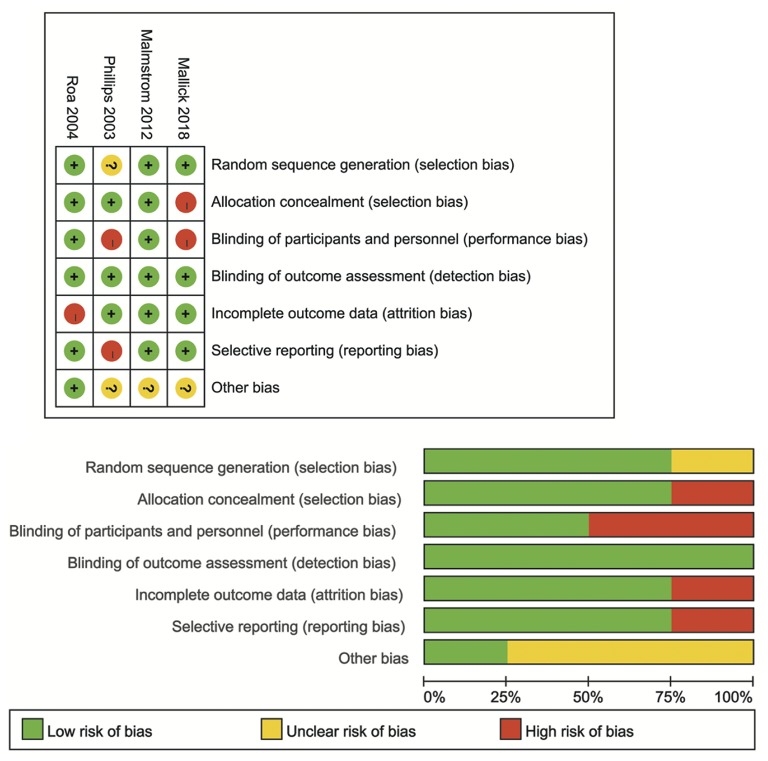
Risk of bias assessment.

The flowchart of study selection is shown in Figure 1 total of 866 articles were identified by the initial search of the electronic databases. Duplicate references (*N* = 392) were removed using the NoteExpress (Aiqing hai, Beijing, Software). The remaining references were evaluated by screening the tiles and abstracts, and 42 articles were selected for screening of the full text. Sixteen studies that the inclusion criteria were included for systematic review (15, 22–37), four RCTs that compared the efficacy and safety of HFR and CFR were included in the meta-analysis (23–26). Of the 16 studies, there were two studies from the same cohort of patients (28, 29). The year of publication ranged from 2003 to 2018. The median OS ranged from 5.1 to 25.2 months. The median PFS was varied from 8.7 to 14.1 month. The information of each included studies are shown in Table 1. The risk of bias assessment for RCTs comparing of HFR and CFR are shown in Figure 2.

**Table 1:** The basic information of included studies for systematic review and meta-analysis.

**Study**	**Year**	**Study Design**	**Cases**	**HFR dose**	**Systemic therapy with HFR**	**Median followed-up (M)**	**Median OS**	**Median PFS**	**Toxicity, QOL, and neurocognitive function**
Navarria et al. (15)	2018	Phase II	30	3.5 Gy ×15	CAAT	8 M	8 M	5 M	The neurologic status remained stable, Grade 2–3 fatigue was observed in 3 patients.
Shenouda et al. (22)	2016	Phase II		3 Gy ×20	Neo-adjuvant TMZ and CAAT	44 M	22.3 M	13.7 M	Grade 5 pancytopenia occurred in one patient, Grade 4 hepatic toxicity was observed in one patient,Grade 3 toxicities consisted of fatigue in 4, nausea and vomiting in 3, and electrolytes imbalances in 3 patients.
Mallick et al. (23)	2018	Phase II RCT	89	3 Gy ×20 vs. 2 Gy ×30	CAAT	11.4 M	25.18 vs. 18.07 M	The entire cohort was 13.5 M	Only two patients required hospital admission for features of raised intracranial tension. One patient in the HFR arm required treatment interruption.
Malmstrom et al. (24)	2012	Phase III,RCT	342	3.4 Gy ×10 vs. 2 Gy ×30 vs. TMZ	-	6 M	7.5 M vs. 6.0 M vs. 8.3 M	-	Nausea and vomiting and hematological toxic effects were more frequently seen in patients treated with TMZ than in those treated with radiotherapy. Grade 3–5 infections were similar among patients in each group.
Phillips et al. (25)	2003	Phase II RCT	68	3.5 Gy ×10 vs. 2 Gy ×30	-	2-monthly	OS:8.7 M vs. 10.3 M	Similar to OS	No late toxicity. No formal QOL comparison was possible as only 30% of patients completed a form.
Roa et al. (26)	2004	RCT, Phase III	95	2.67 Gy ×15 vs. 2 Gy ×30	-	-	5.6 vs. 5.1 M	-	Karnofsky performance status were similar. The rate of Functional Assessment of Cancer Therapy-Brain were too low to performed a meaning comparison.
Floyd et al. (27)	2004	Phase II	18	5 Gy ×10	-	Every 3 M	7 M	6 M	Three patients with brain necrosis (16.7%).
Reddy et al. (28, 29)	2012	Phase II	24	6 Gy ×10	CAAT	14.8 M	16.6 M	-	Significant improvement in insomnia, future uncertainty, motor dysfunction, and drowsiness. Significant worsening was observed in cognitive functioning, social functioning, appetite loss and communication deficit.
Hulshof et al. (30)	2000	Phase II	155	5 ×8 vs. 2 Gy ×33 7 Gy ×4	-	-	5.6 M vs. 7 M vs. 6.6 M	-	No toxicity were observed.
Omuro et al. (31)	2014	Phase II	40	6 Gy ×6	CAAT	42 M	19 M	10 M	The QOL and neuropsychological test scores were stable.
Roa et al. (32)	2015	RCT, Phase III	98	5 Gy ×5 vs. 2.67 Gy ×15	-	6.3 M	7.9 vs. 6.4 M	4.2 vs. 4.2 M	the QOL between both arms at 4 weeks after treatment and 8 weeks after treatment was similar.
Perry et al. (33)	2017	RCT, Phase III	562	2.67 Gy ×15 alone/plus CAAT	CAAT	17 M	7.6 vs. 9.3 M	3.9 M vs. 5.3 M	QOL was similar in the two groups
Minniti et al. (34)	2012	Phase II	71	2.67 Gy ×15	CAAT	-	12.4 M	6 M	Four patients was worsening of their neurologic status. Grade 3 fatigue in 4 patients and cognitive disability in 1 patient.
Ney et al. (35)	2015	Phase II	30	6 Gy ×10	Combined with TMZ and BEV	15.9 M	16.3 M	14.3 M	cranial wound dehiscence/infection in two patients, sepsis in one patient and sudden death from a presumed seizure in one patient.
Iuchi et al. (36)	2014	Phase II	37	8.5 Gy ×8	CAAT	16.3 M	20 M	-	Radiation necrosis was diagnosed in 20 patients (54.1%).
Scoccianti et al. (37)	2017	Phase II	24	4.5 Gy ×15	CAAT	Every 3 M	15.1 M	8.6 M	Three patients (12.5%) had Grade 3 myelotoxicity, One patient had radionecrosis (4.2%).

*BEV, bevacizumab; CAAT, concurrent and adjuvant temozolomide. HFR, Hypofractionated radiotherapy; M, months; OS, overall survival; PFS, progression free survival; QOL, quality of life; RCT, randomized controlled trial; TMZ, temozolomide*.

### Synthesized Findings

#### Results of Trials of Hypofractionated Radiation for Glioblastoma Multiforme

The results of each trial were summarized in Table 1. A Phase II trial conducted in the Netherlands included 155 patients randomized to receive HFR in doses of 66 Gy in 33 fractions, 40 Gy in 8 fractions, and 28 Gy in 4 fractions, respectively. The OS were comparable among the three arms. No adverse events were reported (30).

The median PFS was 6 months and the median OS was 7 months in a Phase II trial that included 18 patients with GBM, who received 50 Gy in 10 fractions (27).

In a trial conducted in [insert the name of the Nordic country], participants had a median OS of 8.3, 7.5, and 6.0 months in the TMZ group, HFR group (34 Gy in 10 fractions) and CFR group (60 Gy in 30 fractions), respectively (24). In patients aged ≥70 years, the OS was better in the TMZ and HFR groups than in the CFR group.

A RCT by Roa et al. (26), demonstrated non-inferiority of HFR (40 Gy in 15 fractions) compared to CFR (60 Gy in 30 fractions) in elderly patients (aged ≥60 years), with a median OS of 5.6 and 5.1 months in the HFR and CFR groups, respectively.

A RCT by Phillips et al. (25), also demonstrated a comparable median OS in the HFR (35 Gy in 10 fractions) and CFR (35 Gy in 10 fractions) groups of 8.7 and 10.3 months, respectively.

A Phase III RCT demonstrated a median OS in the 25 Gy in 5 fractions group and the 40 Gy in 15 fractions group of 7.9 and 6.4 months, respectively (25). In this trial, there were differences between arms in PFS or quality of life outcomes.

Across the studies that we reviewed, the median OS in participants treated with HFR without TMZ was 5.6–8.7 months, and the median PFS was 3.9–8.7 months (24–27, 30). The incidence of brain necrosis ranged from 0 to 17% in the studies that treatment with HFR without adjuvant TMZ.

The introduction of TMZ led to a new standard radiochemotherapy treatment protocol for patients with newly diagnosed GBM. The median OS was comparable in participants who HFR combined with TMZ compared to those who received CFR combined with TMZ. One phase II trial (28, 29) found that 18 patients treated with 60 Gy in 10 fractions with concurrent adjuvant TMZ, had a median OS of 16.6 months and experienced significant improvements in insomnia, future uncertainty, motor dysfunction, and drowsiness. However, cognitive functioning, communication deficit, and social functioning deteriorated.

Scoccianti et al. (37) found that 40 patients treated with HFR (45 Gy in 10 fractions) with concurrent adjuvant TMZ, had a median OS of 15.1 months, and a median PFS of 8.6 months.

Roa et al., conducted a Phase III trial that compared HFR (40 Gy in 15 fractions) to CFR (25 Gy in 5 fractions). The two arms had similar median OS (6.4 vs. 7.9 months) (32).

Perry et al., conducted a Phase III study with 562 participants, who were randomized to receive HFR (40 Gy in 15 fractions) with concurrent adjuvant TMZ, or HFR alone. The participants treated with chemoradiation had a significantly longer median OS (9.3 months), compared to those treated with radiation alone (7.6 months). The PFS was 5.3 and 3.9 months in the chemoradiation group and radiation group, respectively, and the quality of life was similar in the two arms (33).

The median OS was 8 months, and the median PFS was 5 months, in thirty patients received HFR (52.5 Gy in 15 fractions) with concurrent and adjuvant TMZ chemotherapy (15).

In a Phase II trial participants who received HFR (60 Gy in 10 fractions) combined with bevacizumab had a median OS of 16.3 months (35). In a single arm phase II trials, participants who received neo-adjuvant TMZ and concurrent and adjuvant TMZ with HFR (60 Gy in 20 fractions) had a median OS of 22.3 months, and a median PFS of 13.7 months (22).

## Meta-Analysis of Randomized Controlled Trials Comparing Hypofractionated Radiotherapy and Conventional Fraction Radiotherapy for Treatment of Glioblastoma Multiforme

### Overall Survival

**Figure 3:**
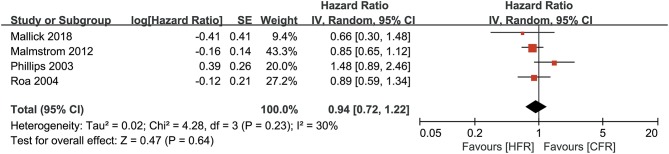
Forest plots for overall survival between hoperfractionated radiotherapy (HFR) and conventional fraction radiotherapy (CFR).

All the RCTs included in the meta-analysis reported OS (26). As shown in Figure 3 participants who received HFR, and participants who received CFR, had a comparable OS (HR: 0.94, 95% CI: 0.72–1.22, *P* = 0.64). A random-effects model was used for this analysis due to moderate heterogeneity (*I*^2^ = 30%, *P* = 0.23).

### Progression-Free Survival

**Figure 4:**
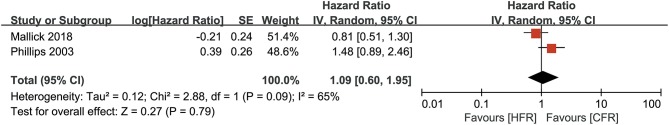
Forest plots for progression-free survival between hoperfractionated radiotherapy (HFR) and conventional fraction radiotherapy (CFR).

Only two studies provided results on the PFS. As shown in Figure 4, pooled data revealed that participants who received HFR, and participants who received CFR chad comparable PFS (HR: 1.09, 95% CI: 0.60–1.95, *P* = 0.79). The analysis was performed using a random-effects model due to high heterogeneity (*I*^2^ = 65%, *P* = 0.09).

### Adverse Events

Adverse events are summarized in Table 2. One trial participant in an HFR group experienced brain necrosis. The overall incidence of complications was similar between the HFR group and CFR group (OR: 1.97, 95% CI: 0.08–49.65, *P* = 0.68). The analysis was performed using a random effects model due to high heterogeneity (*I*^2^ = 89%, *P* = 0.003). There was insufficient data available to compare quality of life between participants who received HFR, and those who received CFR.

**Table 2:** Adverse evens in each group.

**Study**	**Mallick et al**. **(****23****)**		**Malmstrom et al**. **(****24****)**	
**Group**	**HFR**	**CFR**	**HFR**	**CFR**
Radionecrosis	1	0	–	–
‘Hospital admission during RT	2	0	–	–
Steroid requirement after RT	2	0	–	–
RT interruption	1	0	–	–
Deep vein thrombosis	1	1	–	–
Thrombocytopenia grade III/IV	4	0	6	2
Infection	–	–	7	13
Intracranial hemorrhage	–	–	0	3
Vomiting	–	–	1	2
Bleeding	–	–	2	3
Fatigue	–	–	6	6
Nausea	–	–	0	5
Seizures	–	–	7	12

*CFR, conventional fraction radiotherapy; HFR, hoperfractionated radiotherapy*.

### Risk of Bias

#### Sensitivity Analysis and Subgroup Analysis

**Figure 5:**
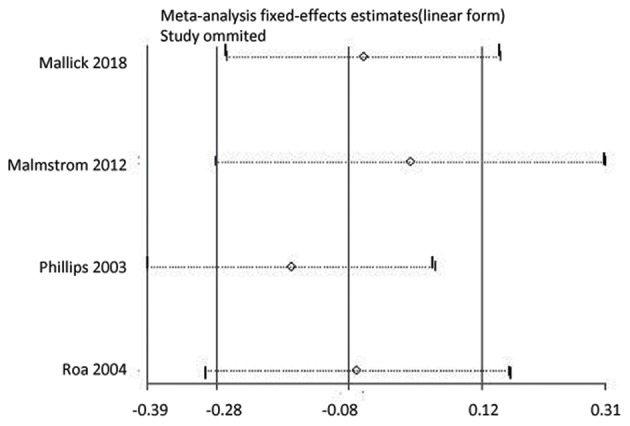
A sensitivity analysis by omitting any single study for overall survival.

We conducted a sensitivity analysis to determine whether the OS results were affected by omitting any single study. As shown in Figure 5, the results were not affected. In addition, we conducted a subgroup analysis comparing OS in participants who received HFR with those who received CFR, stratified by age, using combined data from two studies (24, 26). In participants aged ≥60 years OS was similar in participants who received HFR, compared to those who received CFR (HR: 0.86, 95% CI: 0.69–1.08, *P* = 0.20). However, in participants aged >70 years, participants who received HFR had a longer OS than those who received CFR (HR: 0.59, 95% CI: 0.37–0.93, *P* = 0·02).

#### Publication Bias

**Figure 6:**
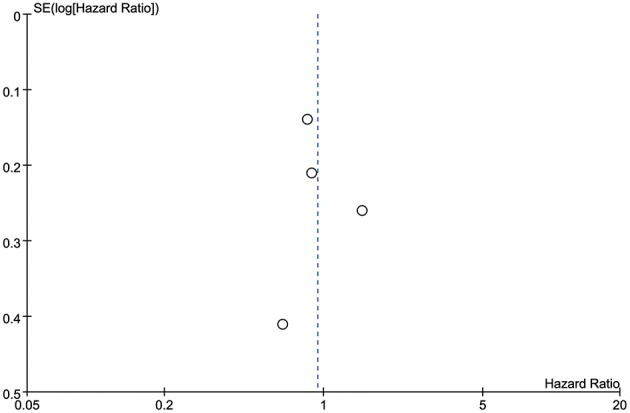
Publication was assessed by funnel plot of overall survival.

Publication bias was evaluated using funnel plots of the OS of each of the studies included in the meta-analysis. All the outcomes were symmetrical within the 95% CIs and no obvious publication bias was observed (Figure 6). Because only four studies were included in the meta-analysis, publication bias was not assessed using Egger's test or Begg's test.

## Discussion

### Summary of Main Findings

Compared to CFR, participants who received HFR had similar OS and PFS, and the incidence of adverse events was comparable between the two treatment modalities. Treatment of GBM is still a challenge in clinical practice. Radiotherapy is considered to be one of the most effective management strategies. However, clinical trials to determine the optimal total dose and fraction doseare ongoing. It is recognized that standard radiochemotherapy is vital for the management of GBM; however, the prognosis of GBM is poor. Almost 90% of local recurrences are located within the irradiation field (38, 39), which suggests that failure to respond to radiotherapy may be attributable to radio-resistance (23). Thus, dose escalation might have some potential benefits in surmounting local recurrence. Either HFR or increasing the total dose could contribute to dose escalation. Early studies have revealed that increasing the total dose to >60 Gy with CFR, using external beam radiotherapy, does not improve survival (40, 41). However, in the trials included in this review, HFR had comparable efficacy and safety to CFR in participants aged ≥70 years.

Some trials have been conducted to assess the optimal total dose and fraction dose for HFR (23–32). A Phase III trial found that the median OS and PFS were 15.1 and 8.6 months, respectively, in participants treated with HFR (either 52.5 Gy in 15 fractions, or 67.5 Gy in 15 fractions) with simultaneous integrated boost plus TMZ (37). The RTOG 9305 trial revealed that providing a stereotactic surgery boost prior to CFR did not produce a survival benefit (42). According to the published studies, the best total dose and fraction dose are not well-determined. Several studies have focused on the strategies of shorten the course of treatment by means of dose escalation (6 Gy × 10, 6 Gy × 6, 8.5 Gy × 8, etc.) (23–36).

Some trials, have evaluated different forms of treatment for GBM. One trial that compared (40 Gy in 15 fractions) to stereotactic surgery (25 Gy in 5 fractions) found that the two groups had comparable OS and PFS (32). The landmark EORTC/NCIC trial found that using CFR plus TMZ, improved OS and PFS compared to CFR alone.

Maximal safe resection, followed by CFR with concomitant and sequential TMZ is considered to be the standard treatment of GBM (37). However, in good performance patients, treatment with HFR could provide some advantages such as reducing the duration and cost of treatment, and the machine time (43).

Some studies have found that participants who received HFR plus TMZ had comparable outcomes to those who received CFR. However, some studies have found that participants who received HFR had a better survival outcomes than those who received CFR (10, 36, 44, 45). A recent meta-analysis based on retrospective studies of the treatment of elderly patients (≥65 years) with GBM, found that participants who received HFR combined with TMZ had similar PFS to participants who received CRF plus TMZ, but that those who received HFR plus TMZ had a shorter OS (46). The results of this study contrast with the results of the study by Malick et al. (23), which was included in our review, which found that participants who received HFR combined with TMZ had a similar survival outcomes to those who received CFR combined with TMZ.

In our subgroup analysis, only two studies evaluated the role of HFR in elderly patients (≥60 years). The results revealed the two radiation regimens had similar OS. In another retrospective study reported by Arvold et al. (8), also suggested that no difference in survival outcomes of CFR plus TMZ and HFR. Minniti et al. (9) reported there were no difference in OS between HFR and CFR. Moreover, PFS were similar between the two groups. More RCTs are required to explore this issue.

In our systematic review and meta-analysis, we did not find a difference in the incidence or profile of adverse events between those treated with HFR and those treated with CFR. However, because of the high heterogeneity between studies, these results should be interpreted with caution. The incidence of adverse events differed from some other published studies. In other studies, the incidence of radiation-induced brain necrosis ranged from 0 to 50% (5, 6, 37, 47). Of the studies included in this review, there was only one case of radiation-induced brain necrosis overall, reported by Mallick et al. (23). The lower incidence of adverse events among participants in the studies included in our review, might be attributable to the new guided imaging delivery technique and a reduction of the target volume margin (from 5 to 3 mm) (23). In regards to quality of life, Roa et al. indicated that HFR (40 Gy in 15 fractions) vs. 25 Gy in 5 fractions had similar quality of life (32). HFR (40 Gy in 15 fractions) with concurrent and adjuvant TMZ vs. this HFR alone also had comparable quality of life (33). Moreover, the neurocognitive function is stable in patients received HFR (15), Minniti et al. reported in 71 patients received HFR, one patient was worse in cognitive function (34). We believe that the introduction of new technology and widely used of proton radiotherapy might greatly reduce the incidence of adverse effects.

### Limitations

This systematic review and meta-analysis has some limitations. Firstly, most of the studies included were Phase II/III trials, and there was a limited number of Phase III trials. This limited the statistical power and the possibility of conducting subgroup analyses. Secondly, in the HFR groups, different total radiation and fraction doses were used, leading to heterogeneity across studies. In addition, some studies compared HFR and CRF both with adjuvant TMZ, while other studies compared HFR and CFR alone. The use of TMZ may have affected the treatment outcomes. Thirdly, there was a lack of studies on patients with young ages and good prognostic factors. Fourthly, the number of studies comparing the quality of life and neurologic functions was limited. Fifthly, improvements in the technology for delivering HFR, may have led to improvements in the efficacy and safety of HFR, some of the findings may be out of date.

## Conclusions

The efficacy and safety of CFR and HFR were similar in terms of survival and the adverse effects of treatment. However, HFR might result to better survival outcomes in patients aged >70 years. The efficacy and safety of HFR for patient with a relatively good prognosis needs to be evaluated further, by means of RCTs. Furthermore, more studies are need to determine the optimal fraction and dose of radiotherapy, and more efforts are required to search for better therapies because the prognosis is poor regardless of the choice of CFR or HFR in patients with GBM.

## Author Contributions

GL and ZZ performed the study search, study selection, data extraction, and analysis. GL, HY, and XL wrote the manuscript. All authors read and approved the final manuscript.

### Conflict of Interest

The authors declare that the research was conducted in the absence of any commercial or financial relationships that could be construed as a potential conflict of interest.
